# Unicentric Castleman disease treated with rituximab before surgery: clinicopathologic findings

**DOI:** 10.1007/s00277-025-06527-3

**Published:** 2025-09-06

**Authors:** Marco Paulli, Giuseppe Neri, Francesca Antoci, Edoardo D’Este, Marco Minetto, Federico Carpi, Martina La Fauci, Marcello Gambacorta, Marco Lucioni, Luca Arcaini

**Affiliations:** 1 Department of Molecular Medicine, University of Pavia, Pavia, Italy; 2 Division of Anatomic Pathology Unit, Fondazione IRCCS Policlinico San Matteo, Pavia, Italy; 3 Pathology Unit, Synlab, Castenedolo, Brescia, Italy; 4 Division of Hematology, Fondazione IRCCS Policlinico San Matteo, Pavia, Italy

**Keywords:** Castleman disease, Histopathology, Rituximab, Microenvironment

## Abstract

Castleman disease (CD) is a rare lymphoproliferative disorder with unique clinicopathological features, including two distinct clinical subtypes categorized as unicentric (UCD) and multicentric (MCD). UCD usually involves a single lymph node site presenting with no or minimal local symptoms. Histologically, most UCD cases exhibit regressive hyaline vascular germinal centers, characterized by penetrating vessels, dendritic hyperplasia/dysplasia, and increased interfollicular vascularity. While complete surgical excision is the standard treatment for UCD, cases treated with neoadjuvant anti-CD20 therapy have been reported. Here, we describe a UCD case treated with anti-CD20, that showed only a partial response. We detail the clinical findings and the histological changes observed in the surgical specimen following therapy, and discuss the potential influence of the local microenvironment on the therapeutic efficacy of anti-CD20.

**Supplementary Information:**

The online version contains supplementary material available at 10.1007/s00277-025-06527-3.

## Introduction

Castleman disease (CD) encompasses a group of rare lymphoproliferative disorders characterized by distinctive clinic-pathological features [1]. CD is broadly classified into two main clinical subtypes: unicentric (UCD) and multicentric disease (MCD). UCD typicaly affects a single lymph node site, with absent/minimal or local symptoms, however 5–15% of patients with hyaline-vascular (HV)-UCD may also present with autoimmune or para-neoplastic conditions such as pemphigus and/or broncholitis obliterans [2–6]. More recently Nishikori et al. [7] described a further distinct subtype of iMCD NOS, termed iMCD -idiopathic plasmacytic lymphadenopathy (iMCD-IPL) [7, 8].

Histopathologic features of CD include the HV/hypervascular (HyperV) subtype, the plasma cell (or plasmacytic) subtype, and mixed forms that are in between [5, 6]. The often striking divergences in terms of clinical presentation require different treatment algorithms [9, 10].

For UCD, the standard treatment for UCD is complete surgical excision [11]. Unicentric masses are typically removed via open surgical procedures, although thoracoscopic or laparoscopy resection are also performed. Video-assisted thoracoscopic surgery (VATS) is rarely used due to the highly vascular nature of these lesions. Other potential UCD treatments include preoperative embolization and radiotherapy [12].

At the best of our knowledge only two previous case reports have described neoadiuvant Rituximab use in UCD [13, 14]. Additionally, front-line Rituximab treatment was reported in a patient initially diagnosed with low grade B-cell lymphoma based on a core needle biopsy (CNB) [15]. Herein we describe the clinicopathologic features of a UCD case treated with Rituximab.

## Case presentation

The patient was a 53-year old man with a history of anxiety managed with benzodiazepines and a former smoker.

In September 2023, he sustained severe polytrauma in a road accident, including a right frontotemporal epidural hematoma, subdural hematoma, and a left frontotemporal coup-contrecoup lacerocontusive focus. The patient reported amnesia for the event and continues to experience a disexecutive syndrome with impaired executive functions, apathetic-abulic behavioral disturbances, and anterograde memory deficits with frequent confabulations.

During the evaluation following the accident, an incidental 10 cm mass, compatible with a gastrointestinal stromal tumor (GIST), was identified. Further evaluations at another hospital in February 2024, including a total-body CT scan, revealed an 89 × 75 mm solid lesion in the jejunum with associated satellite lymphadenopathy along the left external iliac vascular axis, suggestive of a mesenchymal neoplasm.

In the same month, the patient underwent an ultrasonography-guided (UG)-CNB of the abdominal mass. Histological findings suggested CD, possibly of the HV-type.

In early April 2024, blood counts were normal, and HHV-6 and HHV-8 tests were negative. Subsequently, in July 2024, four weekly doses of Rituximab (375 mg e.v.) were administered (off-label treatment) without side effects.

A control PET scan in September 2024 indicated overall metabolic disease stability. The CT scan showed only a slight reduction in the primary mass and satellite adenopathies.

In September 2024, the histological slides from the CNB were re-evaluated at our Pathology Department (MP). Analysis of the specimen revealed needle biopsy fragments, with at least one consisting almost entirely (> 80%) of fibroconnective tissue. The remaining fragments contained lymphoid tissue with widespread and predominant fibro-sclero-hyalinosis, abundant neovascularization, and only rare residual B-cell follicular structures (better identified with anti-MIB1/Ki-67). Immunostaining for immunoglobulin light chains (kappa and lambda) showed sparse plasma cells but provided no definitive diagnostic indications. Immunoreaction with anti-HHV8 was negative throughout the sample. Although a reactive/autoimmune condition including CD, was considered in the differential diagnosis, the biopsy material was deemed insufficient for a conclusive diagnosis. In accordance with the WHO classification [1], a diagnosis of “histological pattern compatible with CD” (major criterion) requires a more adequate biopsy sample for a comprehensive evaluation of the overall architectural pattern.

**Fig. 1:**
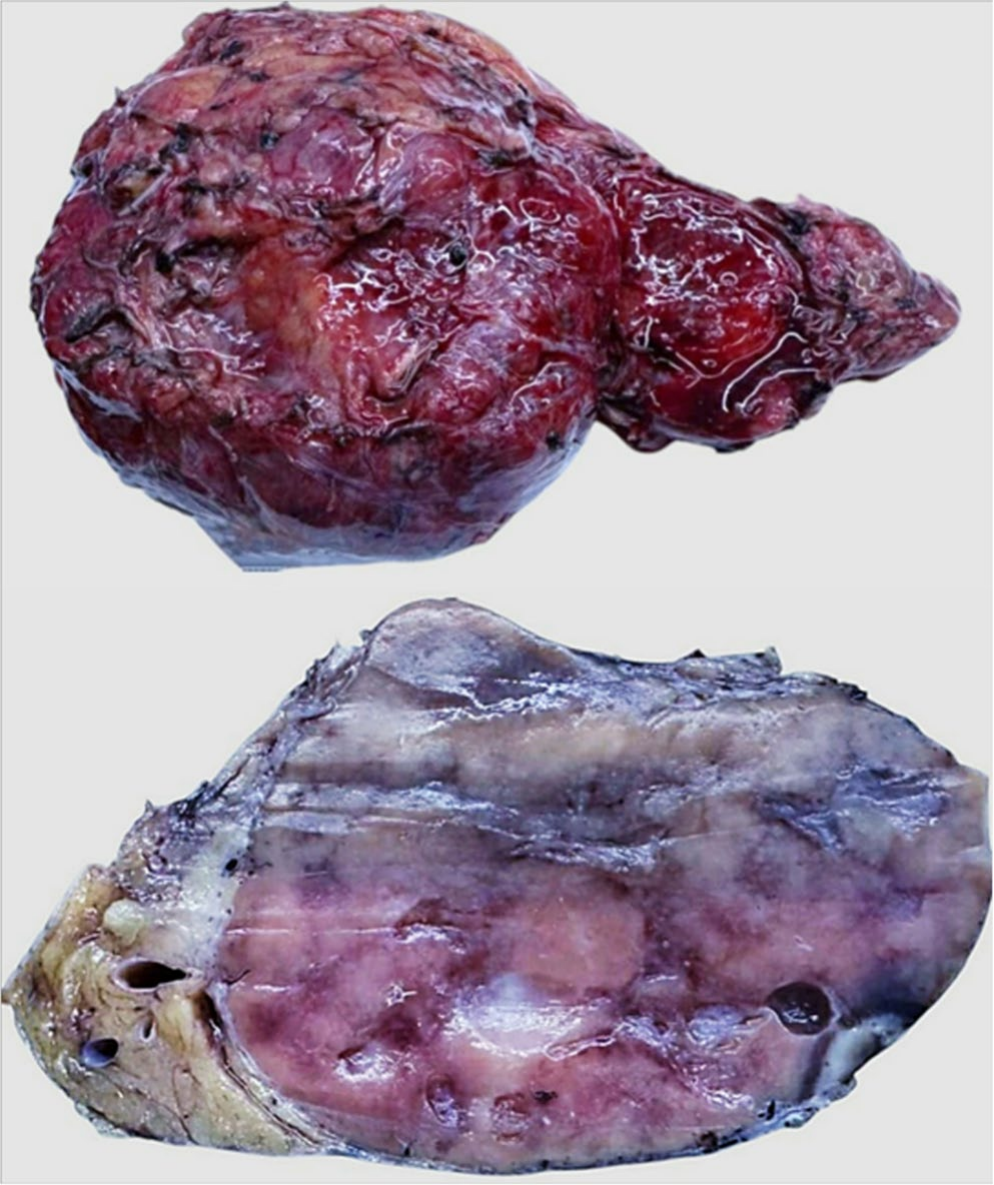
Surgical specimen of patient with unicentric Castleman disease treated with Rituximab

**Fig. 2:**
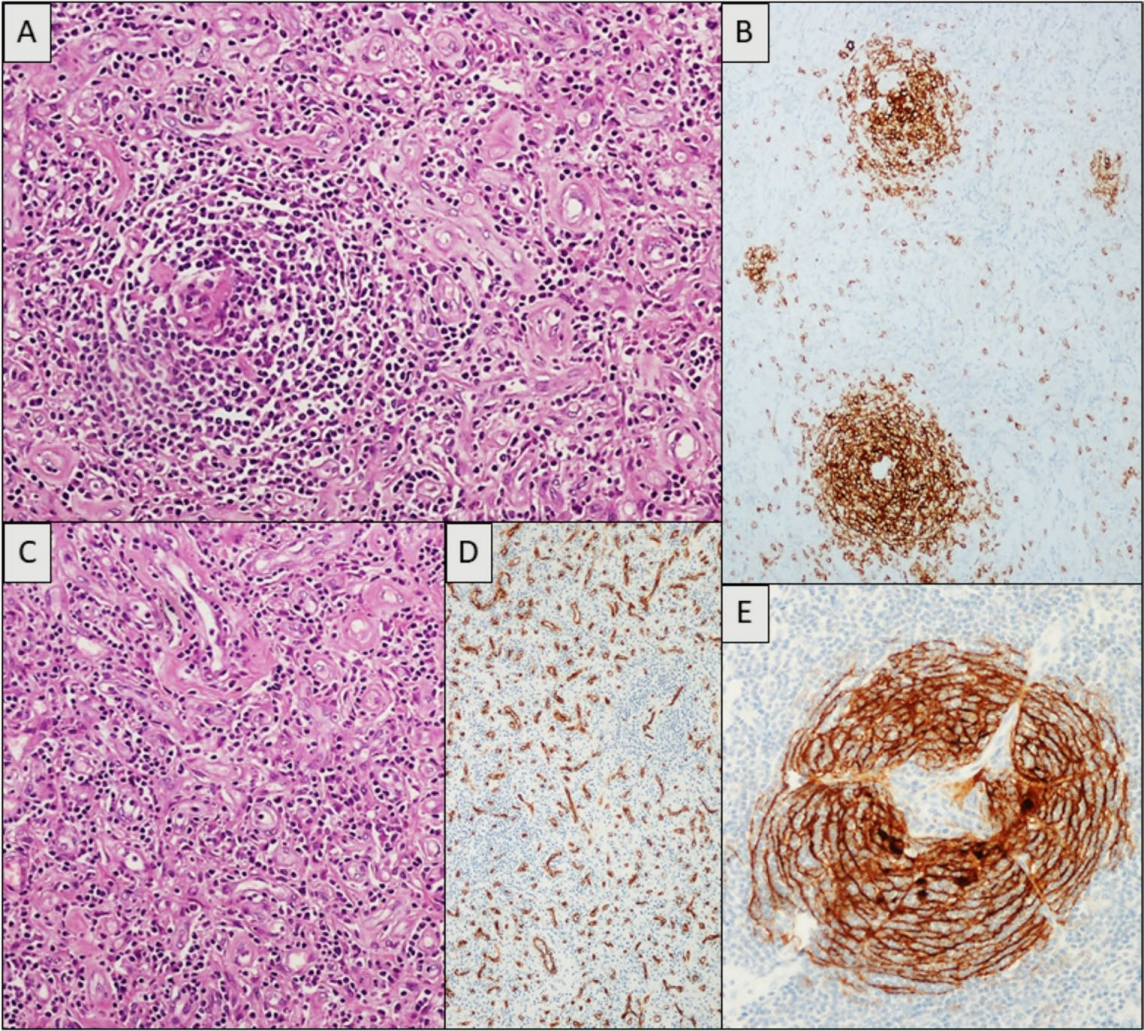
**A-C**) Vascular proliferation adjacent to a follicle with predominantly regressive hyaline vascular changes. **B**) Scattered residual CD20 + B-cell follicles with regressive germinal centers. **C**) Vascular proliferation with prominent endothelial thickening. **D**) The rich vascular component highlighted by anti-CD34. **E)** Dendritic hyperplasia CD21+

On October 2024, the patient underwent laparoscopic biopsy with hand-assisted conversion, followed by complete excision of the lesion. Macroscopically, the surgical specimen (Fig. 1) was a grey to brownish mass measuring 16 × 10.5 × 5.5 cm (fresh measurements). On sectioning, multiple multiloculated formations were observed, the largest measuring 4.5 × 3.5 × 1.5 cm. Microscopically, the lesional tissue predominantly consisted of stromal and vascular proliferation with prominent endothelial thickening and mixed CD3 + T-cell populations. Adipose and fibroconnective tissues were also present, including areas of myxoid mucinous involution forming microcystic spaces. The lymphoid cellular population showed scattered residual CD20 + B-cell follicles with regressive germinal centers, hyalinosis, penetrating vessels, and dendritic hyperplasia (CD21+) (Fig. 2). The CD20 + mantle cell component was significantly, though not completely, reduced (Fig. 2). In conclusion, morphological findings were consistent with CD, HV subtype, with associated very prominent endothelial vascular hyperplasia (Fig. 2). HHV8 was negative. At the most recent check-up in December 2024, the patient was in good condition with no abdominal pain (previous post-surgical tenderness had resolved).

No hepatosplenomegaly or superficial lymphadenopathy was observed. Laboratory tests (December 2024) are reported in the Supplemental Material. A recent CT scan of the neck, chest, and abdomen showed no new solid focal nodular alterations, ground-glass opacities, or suspicious consolidations; no pathological lymphadenopathy was detected in thoracic-mediastinal, axillary, or abdominal-pelvic stations. Considering the re-evaluation data, the patient was started on a periodic follow-up program.

## Discussion

Complete surgical excision remains the gold standard therapy for most UCD cases, leading to an overall survival rate exceeding 90% at five years. The efficacy of the surgical approach was originally described by Castleman himself and recently reaffirmed by evidence-based international consensus guidelines [6, 9, 11].

Rarely, UCD may be unresectable due to its size and localization. Management in such cases is influenced by the presence and type of symptoms (compressive versus inflammatory). Asymptomatic, unresectable disease may be monitored if no neighboring structures are threatened by compression, as UCD can remain stable in size or grow very slowly. For symptomatic, unresectable UCD causing compression of adjacent structures, initial treatment with anti-CD20 Rituximab (with or without steroids) to reduce tumor bulk, followed by surgical resection or radiotherapy (if surgery is still not feasible), is a viable approach [14]. Patients with unresectable UCD and inflammatory symptoms are managed with treatments used for iMCD [10].

In our case, despite the patient remaining asymptomatic, the lack of significant mass reduction following anti-CD20 treatment, coupled with the need for a definitive CD diagnosis reinforced the decision for a surgical approach.

Over the past decades imaging guided CNB has become an increasingly accepted front-line method for nodal tissue sampling, particularly in patients unsuitable for surgical excision, including elderly patients, those with comorbidities and patients with deep-seated lesions. A recent Italian study confirmed the diagnostic efficacy of UG-CNB in the work-up of lymphoid lesions, with the highest sensitivity for aggressive B-cell lymphoma (100%) [16]. This series also included a limited number of CD cases identified via UG-CNB. However, as stated by the recent WHO lymphoma Classification [1], surgical excision is mandatory for CD diagnosis, particularly in cases in which CNB reveals inconclusive [15].

Indeed, in our case only the post-operative histological examination of the lesional mass confirmed the diagnosis of UCD with HV histology and revealed regressive changes likely related to therapy, including myxoid mucinous involution, and microcystic formations. The thickness of the CD20 + mantle zone and the size of the follicles were reduced. The germinal centers exhibited increased hyalinization coupled with prominent hyperplastic/dysplastic dendritic follicle center cells meshwork (Fig. 2b).

While these findings align with previously reported histological changes following neoadjuvant Rituximab, we also observed a highly prominent associated vascular proliferation with prominent endothelial thickening and mixed CD3 + T-cell populations. Furthermore, a substantial number of CD20 + B cells remained detectable, suggesting a significant but only partial response to Rituximab.

Increased vascularity with prominent high endothelial cells are typical features of CD occurring both in the HV UCD, as well as in the iMCD, for which a grading system from mildly to moderately increased to very prominent has been proposed [4].

In our case, we found a marked expansion of the interfollicular tissue by a prominent proliferation of vessels, to the extent that only a few residual regressed HV-follicles could be detected. This CD34 + vascular proliferation (Fig. 2) was quantitatively so relevant that it resembled true vascular hyperplasia, akin to high endothelial venules. The co-occurrence of a vascular neoplasm with CD has been previously reported [17], suggesting that the former might develop from the background of vascular hyperplasia, possibly mediated by angiogenic factors released by the local microenvironment. It seems reasonable that a similar mechanism could be responsible for the vascular proliferation observed in our case.

The anti-tumor action of anti-CD20 antibodies results from the triggering of indirect effector mechanisms of the immune system, including complement-dependent cytotoxicity, antibody-dependent cellular cytotoxicity, or phagocytosis. Despite extensive data on cytotoxicity accumulated over the last two decades, their relative contribution to therapeutic outcome remains difficult to predict in individual cases. Similarly, how anti-CD20 antibodies interact with stromal cells has not been completely clarified. A recent paper [18] suggested that contact with stromal cells could protect tumor cells from anti-CD20 mediated programmed cell death, antibody-dependent cellular phagocytosis, and antibody-dependent cellular cytotoxicity. Proteomic analysis of tumor cells after contact with stromal cells also identified altered pathways, including those involved in cell adhesion, the actin cytoskeleton, and remodeling [19, 20]. As previously described, our patient showed no significant reduction of the lesional mass despite anti-CD20 treatment.

Based on these observations, it could be speculated that the marked stromal and vascular proliferation might, at least in part, be responsible for the persistence of the tumor mass and the reduced effectiveness of the neoadjuvant anti-CD20 treatment.

In these atypical entities, the intranodal expression of CD20 on immunohistochemistry could be a crucial point regarding medical treatment; interestingly Rituximab has been proved to be effective in other atypical lymphadenopathies mimicking lymphoma, such as the progressive transformation of germinal center (PTGC) [21].

## Conclusion

While no definitive conclusions can be drawn from a single observation, further studies on similar cases could help to elucidate the therapeutic implications related to the peculiar local microenvironment associated with CD.

## Supplementary Information

Below is the link to the electronic supplementary material.

Supplementary Material 1

## Data Availability

No datasets were generated or analysed during the current study.
