# Supplementary material for: Unicentric Castleman disease treated with rituximab before surgery: clinicopathologic findings
Source: Ann Hematol. 2025 Sep 6;104(10):5531–5. doi: 10.1007/s00277-025-06527-3 (PMC12619789; doi:10.1007/s00277-025-06527-3)
Supplement: Supplementary file 1 — Supplementary Material 1 [file 277_2025_6527_MOESM1_ESM.docx]

Lab tests (December 2024): hemoglobin 15.5 g/dL, leukocytes 5990/µL, neutrophils 4610/µL, platelets 192,000/µL, normal coagulation tests, fibrinogen 443 mg/dL, normal liver and kidney function, 24-hour proteinuria 0.13 g, BNP 5 ng/L, NT-pro-BNP 36 mg/L, TSH 1.6 mIU/L, vitamin B12 562 pg/mL, folate 19.4 ng/mL, homocysteine 15.1 µmol/L, vitamin D 11.2 ng/mL, bacteriuria in urine analysis, β2-microglobulin 1510 µg/L, ferritin 104 ng/mL, VEGF serum 159.7 pg/mL, VEGF plasma 86.47 pg/mL, IL-6 10.7 pg/mL, normal SPEP, weak IgGκ on serum immunofixation, negative urine immunofixation, κ/λ ratio 1.17.

A
